# Aberrant expression of GSTM5 in lung adenocarcinoma is associated with DNA hypermethylation and poor prognosis

**DOI:** 10.1186/s12885-022-09711-0

**Published:** 2022-06-21

**Authors:** Xuewei Hao, Jun Zhang, Guoyou Chen, Weiwei Cao, Hongyang Chen, Shuo Chen

**Affiliations:** 1 Department of Biochemistry, Inspection Institute, Harbin Medical University-Daqing, Daqing, China; 2 School of Biomedical Sciences and Li Ka Shing Institute of Health Science, The Chinese University of Hong Kong, Hong Kong, China; 3 Department of Biopharmaceutical Sciences, College of Pharmacy, Harbin Medical University-Daqing, No. 39 Xinyang Street, High-tech Zone, Daqing, 163319 Heilongjiang Province China

**Keywords:** Lung adenocarcinoma, GSTM5, DNA methylation, Prognosis

## Abstract

**Background:**

Glutathione-S transferases (GSTs) comprise a series of critical enzymes involved in detoxification of endogenous or xenobiotic compounds. Among several GSTs, Glutathione S-transferases mu (GSTM) has been implicated in a number of cancer types. However, the prognostic value and potential functions of the GSTM family genes have not been investigated in lung adenocarcinoma (LUAD).

**Methods:**

We examined the expression of GSTM5 in LUAD and identified associations among GSTM5 expression, clinicopathological features, survival data from the Cancer Genome Atlas (TCGA). The correlation between GSTM5 DNA methylation and its expression was analyzed using the MEXPRESS tool and UCSC Xena browser. The methylation status of GSTM5 in the promoter region in lung cancer cells was measured by methylation-specific PCR (MSP). After 5-aza-2'-deoxycytidine treatment of lung cancer cells, expression of GSTM5, cell proliferation and migration were assessed by RT-PCR, CCK-8 and transwell assays, respectively.

**Results:**

The results showed that GSTM5 was abnormally down-regulated in LUAD patients’ tissues, and patients with low GSTM5 expression level had significantly shorter OS. Cox regression analyses revealed that GSTM5 was associated with overall survival (OS) of LUAD patients, which expression was an independent prognostic indicator in terms of OS (hazard ratio: 0.848; 95% CI: 0.762–0.945; *P* = 0.003). In addition, we found the promoter region of GSTM5 was hypermethylated in the tumor tissue compared with adjacent normal tissues, and the average methylation level of GSTM5 were moderately correlated with its expression. Moreover, methylation-specific PCR also showed that the GSTM5 gene promoter was hypermethylated in lung cancer cells, and treatment with 5-Aza-CdR can restore the gene expression and inhibit cell proliferation and migration. Finally, Gene Set Enrichment Analysis (GSEA) revealed that low GSTM5 expression was significantly related to DNA repair pathways.

**Conclusion:**

Our data demonstrate that low GSTM5 expression and its high DNA methylation status may act as a novel putative molecular target gene for LUAD.

**Supplementary Information:**

The online version contains supplementary material available at 10.1186/s12885-022-09711-0.

## Background

Lung cancer is the most frequent cause of cancer-related deaths throughout the world [1]. Based on the characteristics and microscopic appearance of the tumor cells, lung cancers are classified into two types, small cell lung cancer (SCLC) and non-small cell lung cancer (NSCLC), the former accounting for approximately 20% of patients and the latter 80% [2–4]. As a type of lung cancer, NSCLC is also subdivided into three histologic subtypes, including lung adenocarcinoma (LUAD), lung squamous cell carcinoma (LUSC) and large-cell carcinoma (LCC). The predominant NSCLC histological phenotype is LUAD, which comprises around 40% of all lung cancer [4–6]. Due to a lack of effective methods for detection of LUAD, the 5-year survival rate for the cancer remains poor. Therefore, it is essential to develop specific biomarkers for the early diagnosis of LUAD.

The glutathione S-transferase (GST) gene family is one of the major xenobiotic detoxifying enzymes that protect cells from toxic drugs and environmental electrophiles [7, 8]. The mammalian GST family consists of three categories: cytosolic, mitochondrial, and membrane-bound microsomal GSTs. Human cytosolic GSTs are divided into seven classes: alpha (α), mu (μ), pi (π), theta (θ), sigma (σ), zeta (ζ) and omega (Ω) [9, 10]. The GSTM gene family (GSTM 1–5) belongs to the cytosolic GSTs, which has been found to confer risk for many cancers. Previous study has been reported that GSTM1 expression was decreased in hepatocellular carcinoma (HCC), and it can exert a tumor-promoting (in MHCC-97H cell line) or tumor-suppressing (in SMMC-7721 cell line) effect by disrupting the ROS-TP53 axis [11]. A study from Kresovich, J.K. et al. showed there was a relationship correlation between GSTM2 promoter hypermethylation and ER/PR-negative tumors in ductal carcinoma in situ and invasive tissue [12]. GSTM5 has also been found to be dysregulated in a variety of tumors, such as Barrett's adenocarcinoma [13], low-stage non-small cell lung cancer [14], ovarian carcinoma [15]. However, the correlation between GSTM family and the prognosis of patients with LUAD remains unknown.

DNA methylation is an important epigenetic modification, which plays a significant role in gene silencing, tissue differentiation, cellular development, X-chromosome inactivation, or genetic imprinting [16–19]. Recently, many studies have revealed that the relationship between methylation level and GSTM gene family expression. For example, Zhao J et al. have reported that high GSTM1 expression due to the hypomethylation of its promoter region is associated with ovarian endometriosis [20]. In addition, previous research also revealed that aberrant DNA methylation in the promoter of GSTM3 reduces the gene expression in age-related cataract lens tissues [21]. However, the relationship between GSTM family expression and gene methylation in LUAD has not been well explored.

In this study, we firstly used online databases to determine the expression profile of GSTM gene family in LUAD patients. Then, we identified the GSTM5 mutation and methylation status and examined its prognostic value in LUAD. Furthermore, the relationship between the methylation status of GSTM5 promoter and the gene expression in lung cancer cells was investigated. Finally, the gene set enrichment analysis (GSEA) was also applied to elucidate the molecular mechanisms of GSTM5 in the LUAD development. Our results demonstrated that the GSTM5 expression was down-regulated in LUAD patients’ tissues, and its expression was affected by methylation, providing a new clue for the diagnosis and treatment of LUAD.

## Methods

### Analysis of Oncomine data and UCSC Xena

The Oncomine database (https://www.oncomine.org) was applied to explore the expression pattern of GSTM gene family in both multiple cancers and their corresponding normal tissues. The expression level of GSTM5 and DNA methylation in patients with primary LUAD were further verified and analyzed using the UCSC Xena browser (http://xena.ucsc.edu/).

### UALCAN database

UALCAN (http://ualcan.path.uab.edu/), an online database for cancer gene expression, allows researchers to analyze transcriptional expression of potential genes of interest between cancer tissues and their corresponding adjacent normal samples and relative clinicopathologic parameters. In this study, we analyzed the expression of GSTM5 in LUAD and its correlations with clinicopathologic parameters through the UALCAN database.

### Gene Set Enrichment Analysis (GSEA)

To investigate the molecular pathways associated with GSTM5 expression in LUAD, GSEA was performed using the GSEA software (https://www.broadinstitute.org/gsea/). Gene expression profiles of LUAD patients were divided into two groups (high expression group and low expression group) according to the median value of expression of GSTM5. Significantly genesets were confirmed with nominal *P-*value < 0.05 and false discovery rates (FDR) < 0.25 after performing 1,000 permutations.

### Cell culture and treatment

Lung cancer A549 cell line and control cell line HPASMC were obtained from the Chinese Academy of Sciences (Shanghai, China). Cells were maintained in RPMI-1640 medium (Invitrogen, USA) supplemented with 10% FBS (Gibco, USA), and grown at 37℃ in a humidified atmosphere containing 5% CO_2_. Treatment of the LUAD cancer cells with or without demethylating agent 5-aza-2’-deoxycytidine (5-Aza-CdR, 5 μmol/L, Sigma) was performed as previously reports. After the above process, the DNA was extracted according to the manufacturer's instructions.

### Methylation-specific PCR (MSP)

DNA methylation patterns at the CpG islands of the GSTM5 promoter were predicted using MethPrimer 2.0 (http://www.urogene.org/methprimer2/). Genomic DNA was extracted according to the instructions of the genomic DNA extraction kit. Then, the DNA was treated with sodium bisulfite, followed by MSP. The methylated and unmethylated GSTM5 were amplified using the following primers: The methylated primers of GSTM5 were: 5’- GGATTTATGGAGTTTTAGGGC-3’ (forward), 5’- ATTCCGAAAACGAAATCAAAACG -3’ (reverse); the unmethylated primers of GSTM5 were: 5’-TGGATTTATGGAGTTTTAGGGT-3’ (forward), 5’- ATTCCAAAAACAAAATCAAAACA -3’ (reverse). The amplified product was separated on a 2% agarose gel and visualized under UV illumination.

### RT-PCR

Total RNA was isolated from cells by Trizol reagent (Invitrogen, Carlsbad, CA) and reverse transcribed to cDNA using reverse transcriptase (TaKaRa). Two pairs of primers for GSTM5 and β-actin were designed according to the gene sequences in GeneBank, and the cDNAs were used as templates for PCR. The GSTM5 primers were: 5’- ATGCCCATGACACTGGGGTACTG-3’ (forward), 5’- CCATGTGGTTATCCATAACCTGG-3’ (reverse) [22]. The β-actin primers were: 5’- ACTATCGGCAATGAGCG -3’ (forward), 5’- GAGCCAGGGCAGTAATCT -3’ (reverse). Amplified PCR products were separated in 1.5% agarose gel electrophoresis.

### Cell viability assay

The Cell Counting Kit-8 assay was performed to measure cell viability according to manufacturer’s instruction. The A549 cells were placed on 96-well plates, then the cells were treated with different methods. A total of 100μL CCK-8 reagent was added to each well and the cells were incubated for further 4 h. The cell viability was evaluated at the absorbance of 450 nm by using a microplate reader.

### *In vitro* transwell assay

Cell activity was measured through polycarbonate membrane Boyden chambers in a transwell apparatus (Corning, NY, USA). Treated cells were seeded into the top chamber and 500 μL of RPMI 1640 containing 10% FBS was added to the bottom chamber. After 24 h of incubation at 37˚C, cells in the lower chamber were fixed and then stained with 0.2% crystal violet. The Leica DMI3000B microscope (Leica, Wetzlar, Germany) was performed to calculate the number of migrated cells.

### Statistical analyses

All statistical analyses were performed using SPSS 25.0 (IBM, NY, USA) and GraphPad Prism 8,0 (GraphPad Software, CA, USA). The Welch’s t-test was used to analyze the difference in gene expression between groups. The log-rank test was used to examine the differences between the survival curves. The prognostic value of gene expression was analyzed using the univariate and multivariate Cox regression models. Association between GSTM5 and the clinicopathological parameters in LUAD patients was examined using χ^2^ test with a two-sided Fisher's exact test. *P* < 0.05 was considered to be statistically significant.

## Results

### GSTM gene family transcript expression in LUAD

**Fig. 1:**
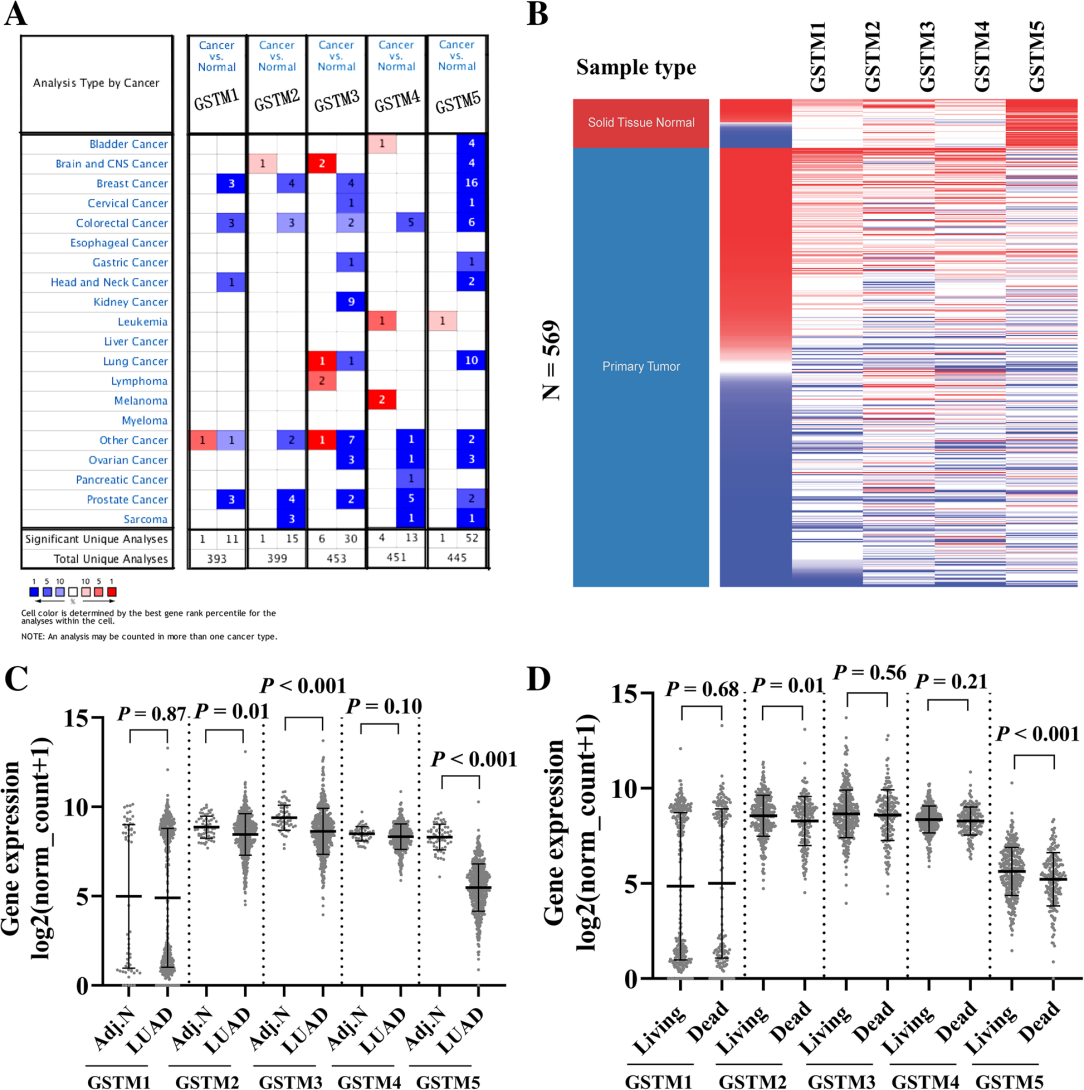
Expression profiles of GSTM family members in LUAD patients. **A** GSTM family genes expression in various tumors from Oncomine database. **B** Heatmap showing expression of GSTM family genes in LUAD. **C** Plots chart comparing the expression of GSTM family genes in LUAD and matched adjacent normal tissues. **D** The expression profiles of the GSTM gene family according to their OS status

The expression profiles of GSTM gene family were identified using the Oncomine database. Results revealed that GSTM3, GSTM5 expression was down-regulated in lung tissues compared with their matched normal tissues (Fig. 1A). Raw data and clinical information were downloaded from UCSC Xena websites. After filtering out the samples with incomplete follow-up information, a total of 511 LUAD samples and 58 normal samples were obtained for further analysis. By comparing the expression of GSTM gene family with adjacent normal tissues, we found that the expression GSTM2, GSTM3 and GSTM5 was significantly decreased, whereas GSTM1 and GSTM4 showed no significantly difference (Fig. 1B and C). We also comparted the expression profiles of the GSTM gene family according to their OS status, and observed GSTM2 and GSTM5 expression were decreased in the dead group (Fig. 1D) (for more basic data, please see additional file 1).

### GSTM5 acted as a putative prognostic factor in LUAD

**Fig. 2:**
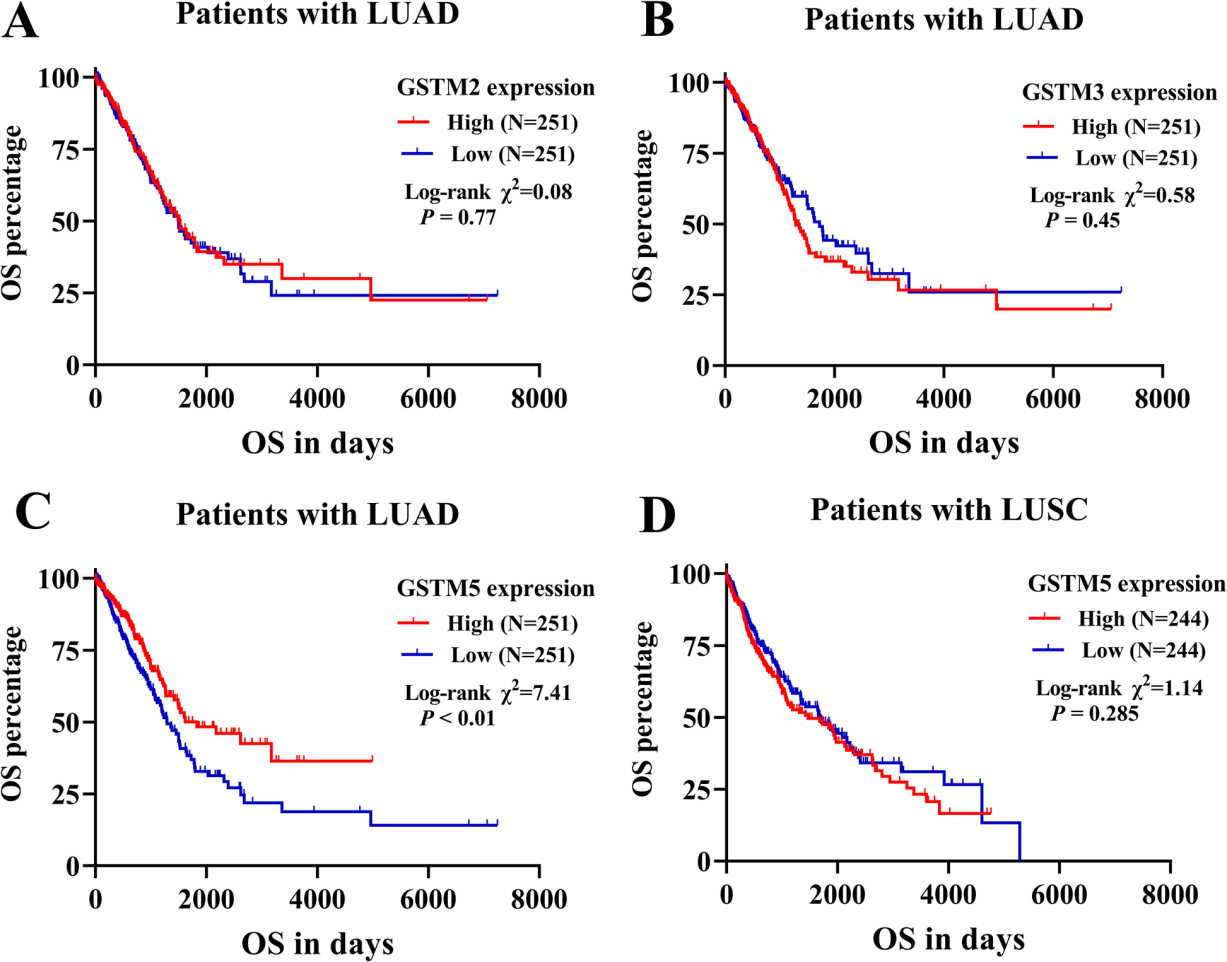
The Kaplan–Meier survival curves of OS in LUAD and LUSC patients. LUAD patients were grouped according to the GSTM2 (**A**), GSTM3 (**B**) and GSTM5 (**C**) expression in ROC analysis for OS detection. (**D**) The association between GSTM5 expression and OS in LUSC patients

**Fig. 3:**
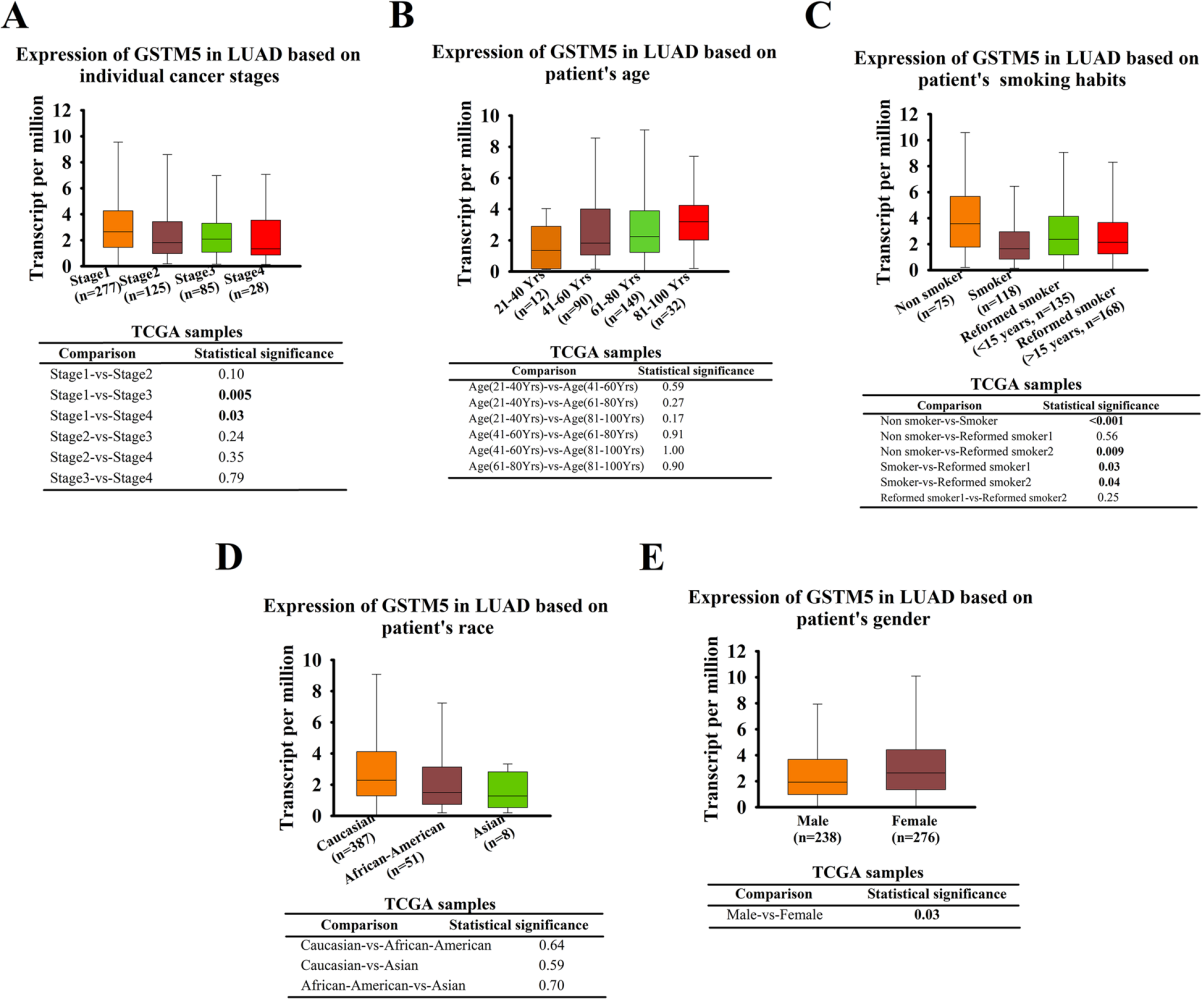
Association between GSTM5 expression and clinical characteristics in LUAD patients (UALCAN database). Comparison of GSTM5 expression according to individual cancer stages (**A**), patient's age (**B**), smoking habits (**C**), patient's race (**D**), patient's gender (**E**)

To further examine the prognostic potential of these 3 dysregulated genes in LUAD, the Kaplan–Meier plotter was utilized to evaluate the association between 3 GSTMs and OS. Results of log-rank test demonstrated that the low expressions of GSTM5 was associated with poor OS, while the expression of GSTM2 and GSTM3 showed no statistically significant relationship to patient OS (Fig. 2A, B and C). In comparison, there was no significant association between GSTM5 expression and patient OS in LUSC (Fig. 2D) (for more basic data, please see additional file 2). We also scrutinized the UALCAN tool to determine whether there is an association between GSTM5 expression in LUAD tissues and their clinicopathologic parameters, such as clinical stage, patient’s gender, age, race, stage, and smoking habits, and the results were shown in (Fig. 3A-E). We performed univariate and multivariate analyses to further assess the prognostic value of GSTM5. Univariate analysis showed that pathological stage, radiation therapy, and GSTM5 expression were risk factors of shorter OS (hazard ratio: 0.848; 95% CI: 0.762–0.945; *P* = 0.003). Multivariate analysis showed that GSTM5 expression an independent prognostic marker for LUAD (hazard ratio: 0.884; 95% CI: 0.794–0.981; *P* = 0.023) (Table 1) (for more basic data, please see additional file 3). Taken together, these results indicate that there is a significant positive association between GSTM5 expression and LUAD aggressiveness.

**Table 1:** Univariate and multivariate analysis of the prognostic value of GSTM5 expression regarding OS in patients with LUAD

Parameters	Univariate analysis				Multivariate analysis			
	*P*	HR	95%Cl (lower/upper)		*P*	HR	95%Cl (lower/upper)	
Age	0.307	1.008	0.993	1.024				
Gender								
Female *vs.* Male	0.842	0.971	0.724	1.302				
Pathological stage								
I/II *vs.* III/IV	**< 0.001**	0.384	0.281	0.526				
Targeted molecular therapy								
Yes *vs.* No	0.410	0.869	0.621	1.214				
Smoking History								
Yes *vs.* No	0.617	0.900	0.596	1.360				
Radiation therapy								
Yes *vs.* No	**< 0.001**	0.500	0.340	0.735				
GSTM5 expression	**0.003**	0.848	0.762	0.945	**0.023**	0.884	0.794	0.981
Longest tumor dimension	0.929	0.983	0.681	1.421				

*Note*: Multivariate analysis was performed by setting pathological stages, radiation therapy and GSTM5 expression as covariates

Bold indicates *P*< 0.05

### GSTM5 expression was negatively correlated with its methylation

**Fig. 4:**
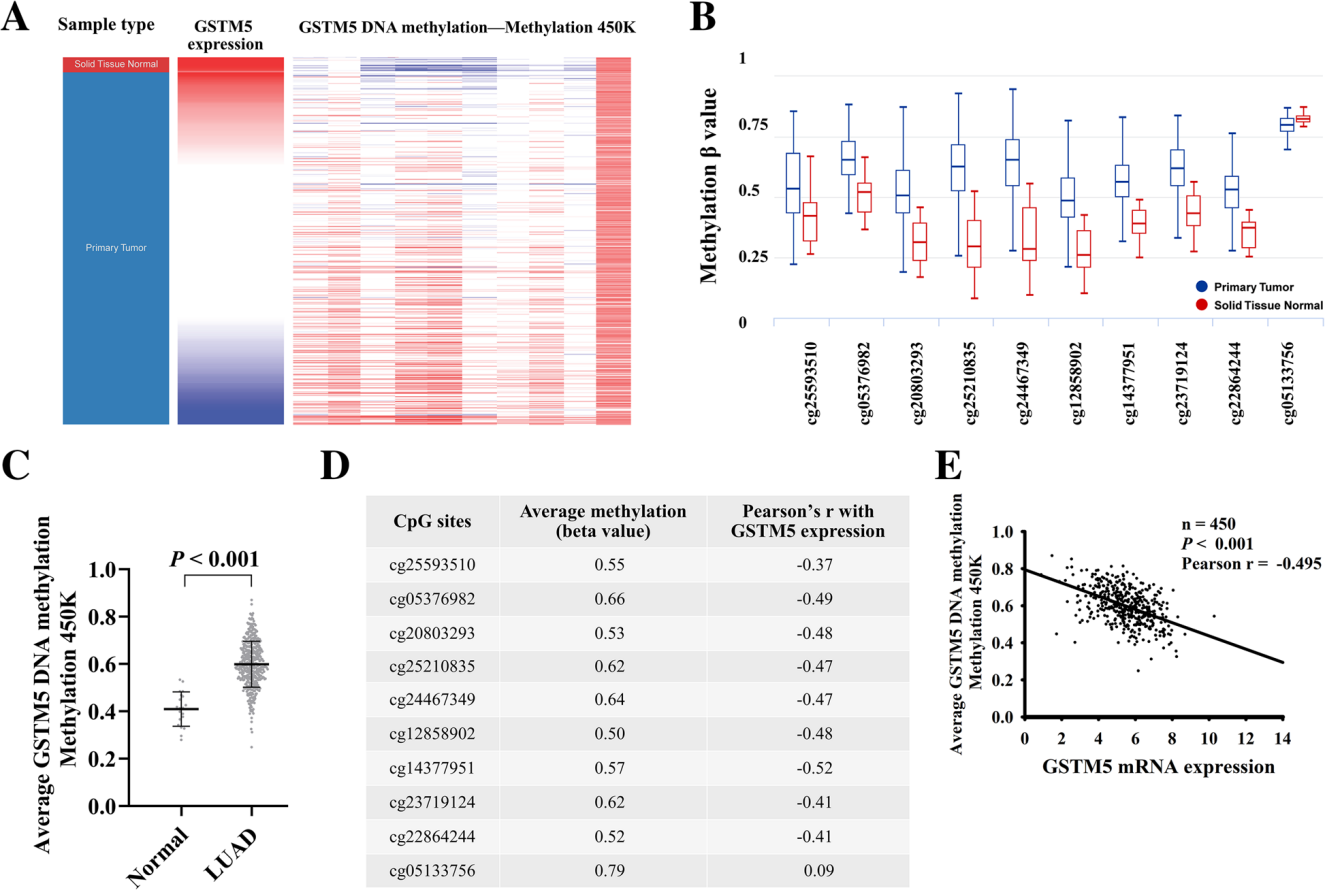
GSTM5 expression and its DNA methylation status in LUAD. **A** Heat map of GSTM5 expression and DNA methylation status. **B** Plots chart comparing the GSTM5 methylation level in LUAD and normal tissues. **C** Comparison of the average methylation level of GSTM5 gene between LUAD and matched normal tissues. **D** Correlation analyses between CpG sites methylation frequencies and GSTM5 expression. **E** The correlation between GSTM5 expression and the average DNA methylation in LUAD

**Fig. 5:**
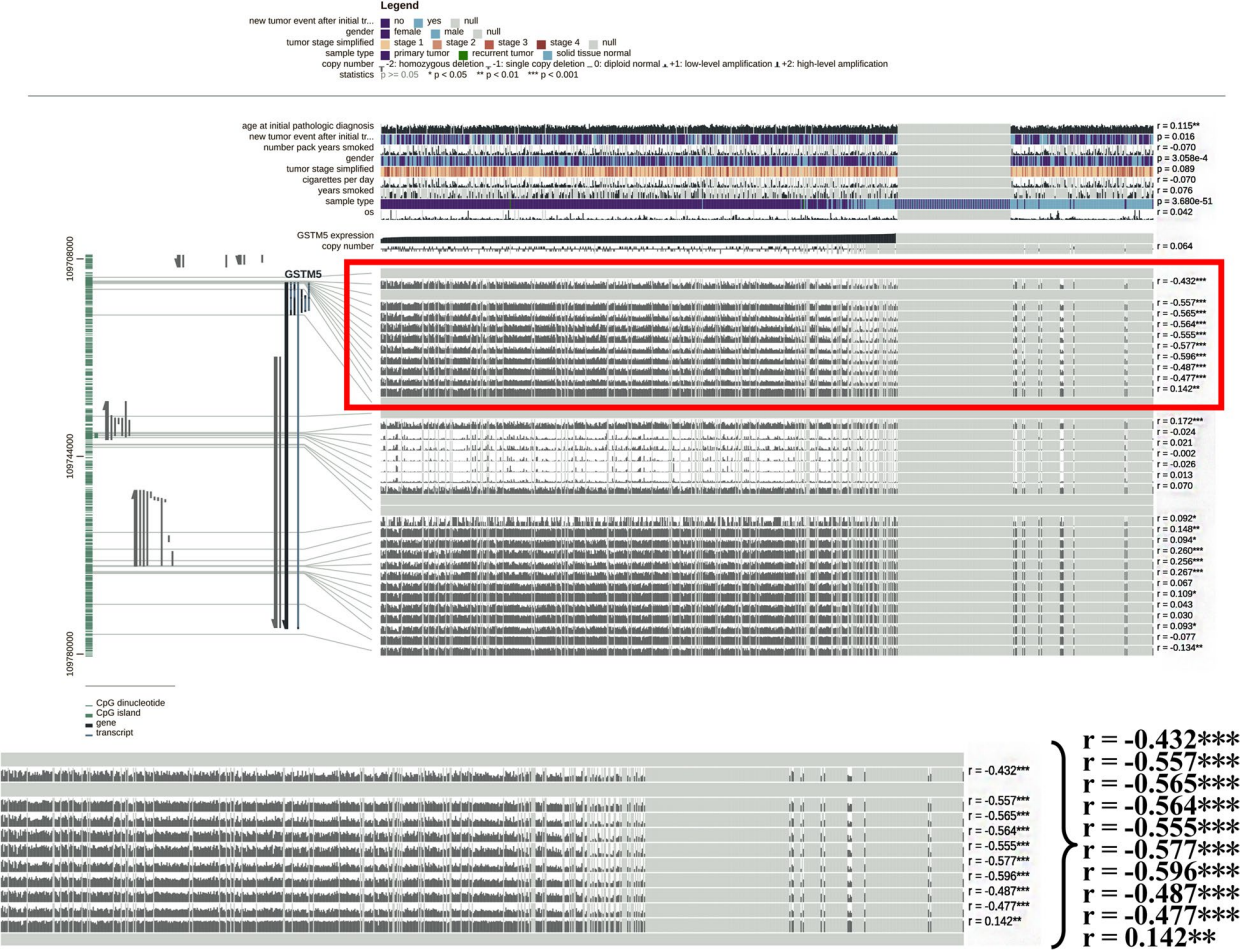
The correlation between GSTM5 expression and its DNA methylation was showed using MEXPRESS

Using the Illumina Methylation 450k Beadchip, we compared the methylation status of 10 CpG sites in GSTM5 DNA between cancer tissues and their adjacent normal samples. Results showed 9 sites in GSTM5 were significantly hypermethylated in the tumor tissue compared with adjacent normal tissues (Fig. 4A-D). In addition, regression analysis demonstrated that the expression level of GSTM5 was inversely correlated with its methylation status (Fig. 4E). To further confirm our results, an association analysis was carried to examine the relationship using MEXPRESS database. The results revealed that the promoter region of GSTM5 showed higher methylation level in LUAD compared to normal tissues and there is a negative correlation between them (highlighted part with red box) (Fig. 5). These findings imply that DNA methylation might be also a molecular mechanism leading to GSTM5 down-regulation.

### 5-Aza-CdR reversed GSTM5 methylation and promoted GSTM5 expression in LUAD cells

**Fig. 6:**
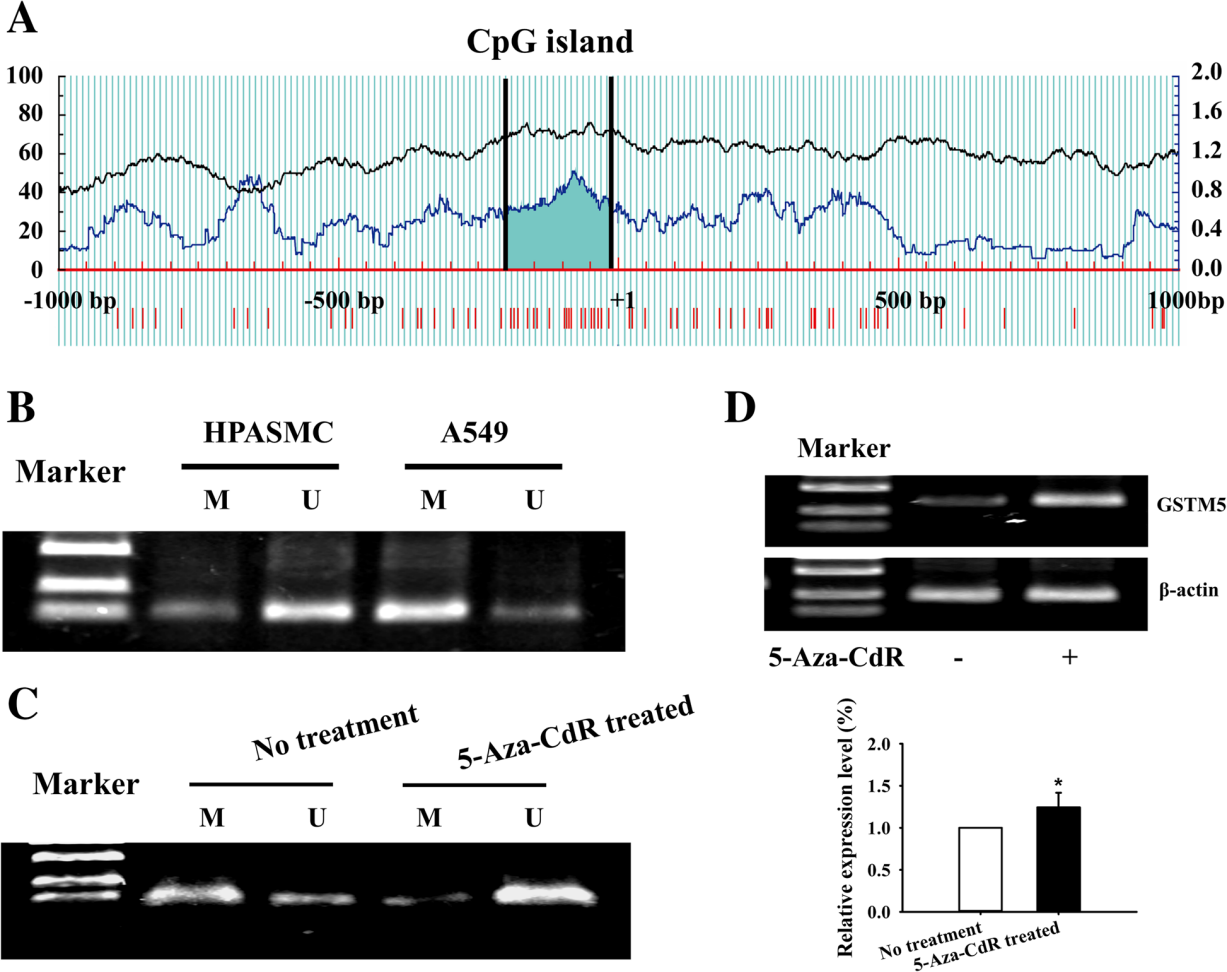
5-Aza-CdR demethylated the GSTM5 promoter region and promoted GSTM5 expression in LUAD cells. **A** The CpG island of GSTM5 was predicted by MethPrimer. **B** Methylation status of the promoter region of GSTM5 in lung cancer cells. **C** Effect of 5-Aza-CdR on GSTM5 demethylation in LUAD cells. **D** GSTM5 mRNA expression analysis by RT-PCR in LUAD cells following 5-Aza-CdR treatment for 48 h. Full length gels and blots are also included in additional file 4. M: amplification product of methylated primers; U: amplification product with non-methylated primers. ^*^P < 0.05

Prediction of GSTM5 promoter methylation was performed through the Methprimer tool. The result showed that there is a high proportion of CpG in the region of GSTM5 promoter, which could provide large numbers of methylation modification sites (Fig. 6A). Methylation-specific PCR results showed that the GSTM5 gene was hyper-methylated in LUAD cancer cell line, suggesting that GSTM5 gene silencing in cancer cell may be associated with the hypermethylation of its promoter (Fig. 6B). Then, we investigated the effects of 5-Aza-CdR on the methylation level and the expression of GSTM5 in lung cancer cell line. The results showed that the GSTM5 gene methylation was reversed by 5-Aza-CdR, and the methylation inhibitor could restore the gene expression in lung cancer cells (Fig. 6C and D). These results suggested that the hypermethylation of GSTM5 promoter region may contribute to the down-regulation of gene expression.

### Effects of 5-Aza-CdR on the cell viability and the cell migration of A549 cells

**Fig. 7:**
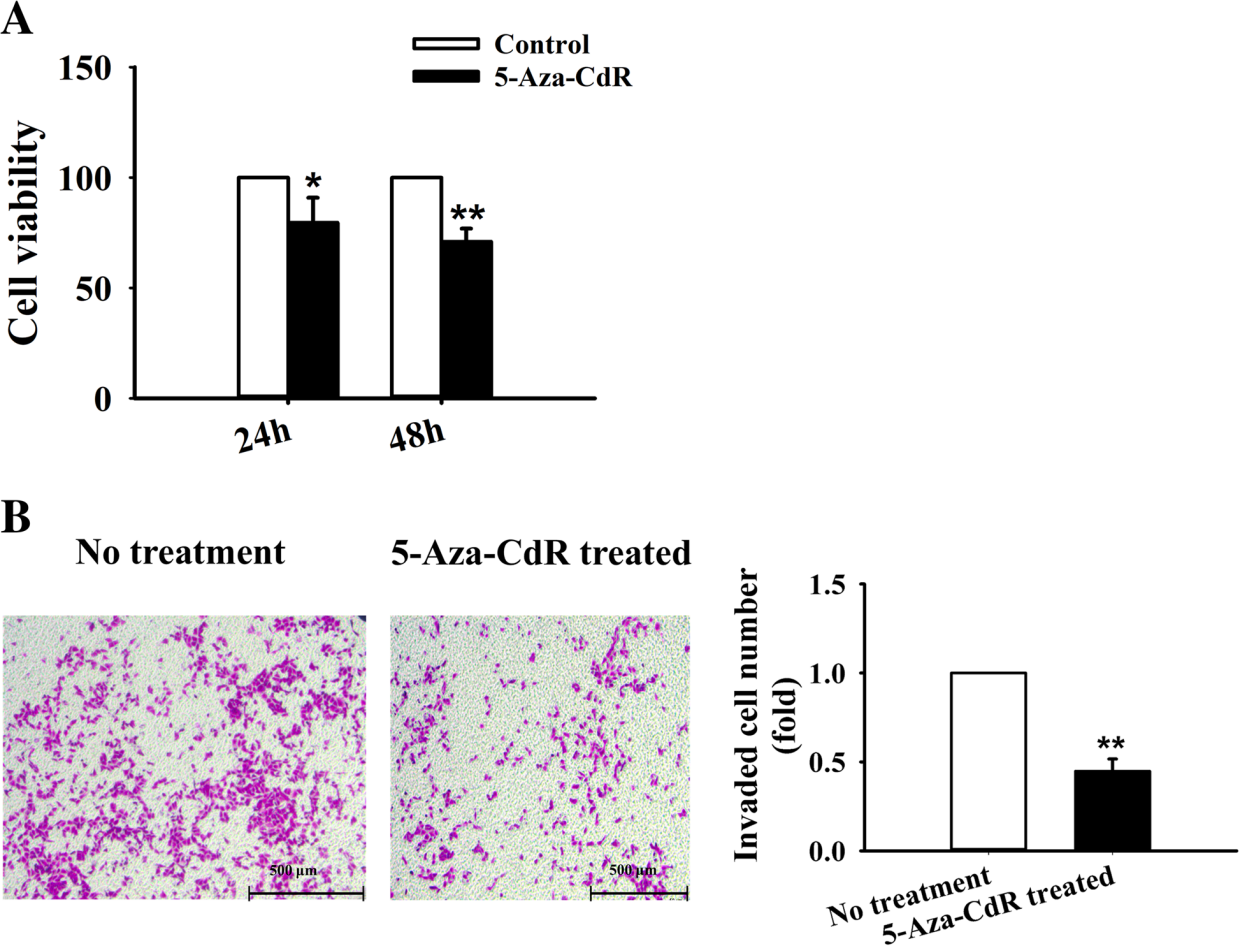
5-Aza-CdR inhibited the proliferation and migration capacity of A549 cells. **A** Cell proliferation was measured by CCK-8 assay after 5-Aza-CdR treatment. **B** Transwell assay was employed to determine cell migration after 5-Aza-CdR treatment (magnification, × 50). ^*^P < 0.05, ^**^P < 0.01

To investigate the cellular effects resulting from 5-AzaCdR, the CCK-8 assay was performed to assess the cell proliferation rate. The result showed that 5 μM 5-AzaCdR significantly restrained the cell proliferation (Fig. 7A). We also used transwell assay to examine the effect of 5-Aza-CdR on lung cancer cell migration, and found that 5-Aza-CdR inhibited the cell motility potential by 55% as compared to control group (Fig. 7B). Based on the obtained results, it was indicated that treatment of A549 cells with 5-Aza-CdR may suppresses the cell proliferation and migration by restoring the GSTM5 expression through reversing the gene promoter region hypermethylation status.

### GSEA identified GSTM5-related signaling pathways

**Fig. 8:**
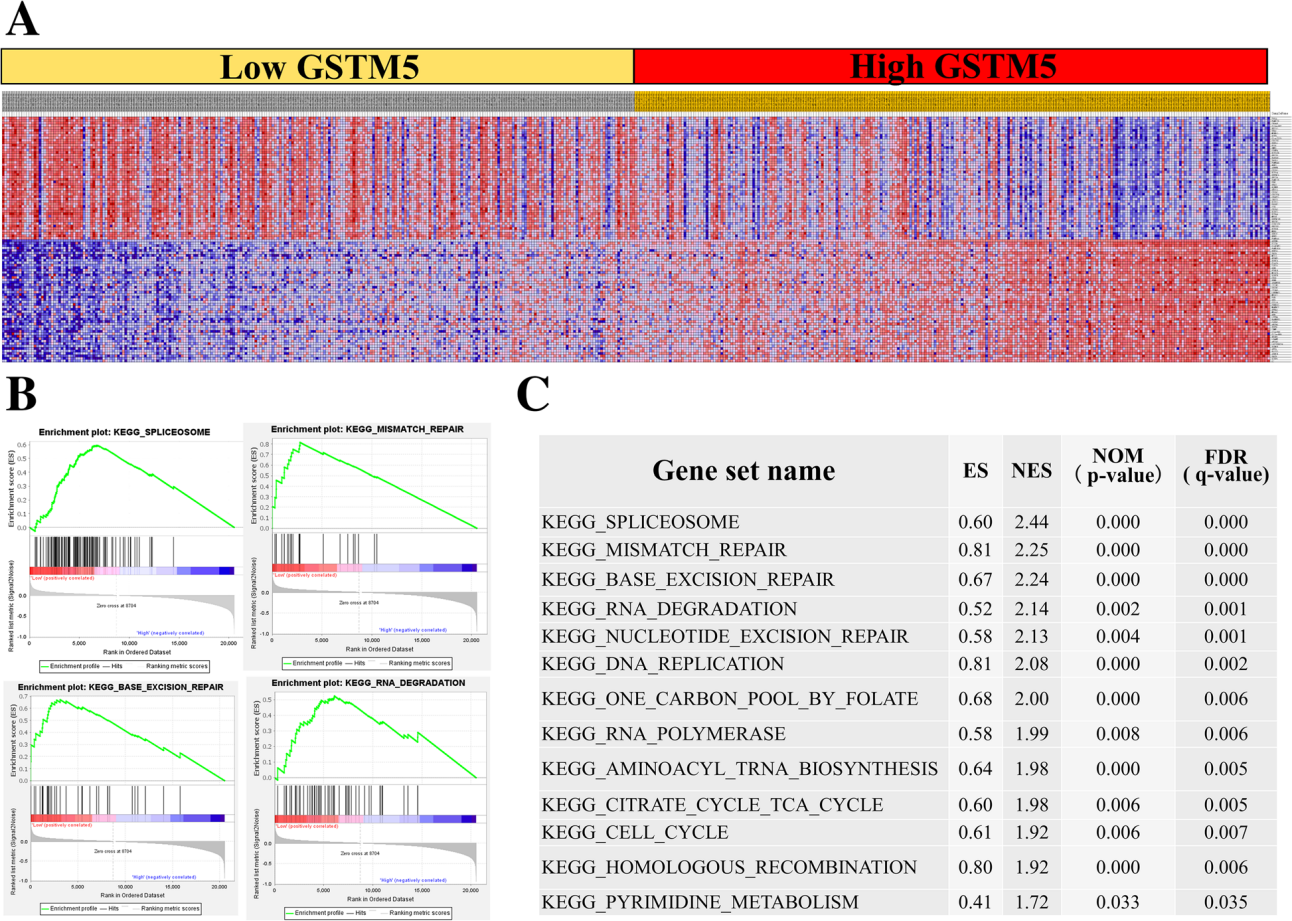
The GSEA analysis showed the potential downstream signaling of GSTM5. **A** Heatmap of the top 50 features for each phenotype in single GSTM5 gene enrichment analysis in TCGA LUAD database. **B** Low GSTM5 expression group was enriched in spliceosome, mismatch repair, base excision repair, RNA degradation gene sets. **C** Gene sets enriched in phenotype of low GSTM5 expression group. ES, enrichment score; NES, normalized ES; NOM *P*-val, normalized *P*-value

To identify the cellular mechanisms by which GSTM5 influences LUAD development, we performed GSEA using TCGA data. Total LUAD samples were divided into two groups by the median expression of GSTM5, namely GSTM5^low^ group and GSTM5^high^ group (Fig. 8A). The analysis revealed that low GSTM5 expression positively associated with DNA repair pathways, such as spliceosome, mismatch repair, base excision repair, RNA degradation gene sets, which suggested that GSTM5 may regulate LUAD cell proliferation through the gene mutation. Figure 8B and C exhibited top 4 pathways correlated with GSTM5 and all gene sets enriched in GSTM5^low^ group.

## Discussion

In this study, with the help of Oncomine and TCGA database, we found that GSTM5 expression was significantly decreased in LUAD tissues compared with matched non-neoplastic tissues, and low GSTM5 expression level was associated with a poor prognosis of LUAD. Second, we found that increased the gene promoter methylation status may be a mechanism of its down-regulation. *In vitro* assays also demonstrated that treatment with the 5-Aza-CdR could restore the gene expression, thus resulting in a decrease of proliferation and migration of the lung cancer cells. Finally, based on GSEA, we predicted the potential mechanisms by which GSTM5 contributes to pathogenesis of LUAD.

The GSTM5 belongs to the Mu class of glutathione-S-transferases (GSTs) family, members of which spans a 97-kb region on chromosome 1p13 [23]. Previous study revealed that GSTM5 expression was down-regulated in bladder cancer tissues than in normal tissues, and overexpression of GSTM5 decreased the bladder cancer cell proliferation, migration and colony formation capacity, suggesting that GSMT5 may act as an effective tumor suppressor in bladder cancer [22]. Furthermore, low expression levels of GSTM5 were also observed in breast cancer, prostate cancer and Barrett's adenocarcinoma [13, 24]. However, it has also been reported that GSTM5 was up-expressed in colon cancer tissues than in corresponding normal tissue. The survival analysis indicated dysregulated GSTM5 expression in colorectal cancer samples was associated with poor survival [25, 26]. Moreover, Yeyang et al. also reported that high GSTM5 expression was associated with a worse prognosis in patients with gastric cancer [27]. Over the last few years, several studies have reported that certain tumor suppressor genes might act as “double agents” with both tumor suppressor and promoter functions [28]. PTEN was recognized as a tumor suppressor of which the expression is often lost in many tumor types [29–31]. Mutation of PTEN is consistently observed in human T-ALL specimens, where loss of PTEN promotes leukemia stem cells growth through activation of PI3K/AKT signaling [32]. However, In B-ALL and CML, deletion of PTEN does not accelerate leukemia cell growth and has the opposite effect [33]. The phenomenon that protein possess both oncogenic and tumor-suppressing activities is not uncommon. For these proteins are involved in many cellular pathways and depend on different cellular contexts, thus leading to a variety of pathological outcomes [34]. In the current study, based on Oncomine database and UCSC database, we found that in LUAD tissues, the expression of GSTM5 was reduced compared with levels in normal controls, and the patients with low GSTM5 expression exhibited worse prognosis and survival than patients with high GSTM5 expression.

Then, we tried to investigate the mechanisms of GSTM5 dysregulation. DNA methylation, one of the most important epigenetic modifications, has been demonstrated to play essential roles in cellular processes such as regulation of gene transcription. Aberrant DNA methylation patterns are generally associated with a range of human diseases, including cancer [35, 36]. Myelodysplastic syndrome (MDS) is clinically heterogeneous disease and tends to transform into acute myeloid leukemia. Refractory anemia with excess blast (RAEB) and refractory cytopenia with multilineage dysplasia (RCMD) are two MDS subgroups linked, and RAEB is the most common pre-leukemic type which has a relatively poor prognosis. Hypermethylation of CpG island of GSTM5 gene was observed more frequently in RAEB patients than in RCMD patients, and the hypermethylation phenotype was positively associated with malignant disease progression, suggesting it has a role in MDS progression [37]. Methylation of the GSTM5 promoter region has also been reported in several malignant tumors, including bladder cancer, Barrett's adenocarcinoma and thyroid carcinoma [13, 22, 38]. Considering these pieces of evidence, we speculated that the hypermethylation of GSTM5 in promoter may be a main mechanism of the gene silencing in LUAD. So, we explored the correlation between GSTM5 expression level and its DNA methylation. Based on the UCSC Xena and MEXPRESS, we found that there was a negative correlation between GSTM5 methylation level and its expression. *In vitro* studies, our study indicated that the promoter region of GSTM5 was methylated in A549 lung cancer cells and GSTM5 gene expression was related to the promoter region methylation status. 5-Aza-CdR is a potent DNA demethylating agent that has been widely used to induce gene re-expression and cellular differentiation. As a cytotoxic agent in proliferating cells, the drug causes multiple changes in cell physiology and is utilized as a potent antitumor agent for cancer therapy [39]. In this study, we found that 5-Aza-CdR treatment could reverse the GSTM5 promoter region hypermethylation status and restore the gene expression, thus resulting in a decrease of proliferation and migration of A549 cells. Therefore, our results suggest that hypermethylation of GSTM5 promoter region might be an important mechanism of dysregulation of GSTM5 and demethylation may restore GSTM5 tumor suppressor activity. To better understand the role of GSTM5 in the regulation of LUAD, GSEA was conducted to explore the gene sets enriched in cancer patients. The analysis showed that low GSTM5 expression was positively associated with related to DNA repair pathways which may suggest that GSTM5 is involved in LUAD progression through these cancer-associated pathways.

## Conclusions

Taken together, we found that the expression level of GSTM5 was significantly downregulated in LUAD and its expression was associated with overall survival of LUAD patients. Furthermore, increased DNA methylation contributed to the aberrant GSTM5 expression. Finally, 5-Aza-CdR caused demethylation and reactivated the GSTM5 gene to restore its anticancer ability. In conclusion, our study showed that GSTM5 may serve as prognostic biomarkers for LUAD patients in the future.

## Supplementary Information

**Additional file 1.**

**Additional file 2.**

**Additional file 3.**

**Additional file 4.**

## Data Availability

The datasets generated and/or analyzed during the current study are available in Oncomine, UCSC Xena, UALCAN, MethPrimer 2.0 [https://www.oncomine.org; http://xena.ucsc.edu/; http://ualcan.path.uab.edu/; http://www.urogene.org/methprimer2/]. All data and outcomes generated during this study are included in this published article.

## Abbreviations

GSTM: Glutathione S-transferases mu
LUAD: Lung adenocarcinoma
TCGA: The Cancer Genome Atlas
GSEA: Gene Set Enrichment Analysis
SCLC: Small cell lung cancer
NSCLC: Non-small cell lung cancer
LUSC: Lung squamous cell carcinoma
LCC: Large-cell carcinoma
GST: Glutathione S-transferase
5-Aza-CdR: 5-Aza-2’-deoxycytidine; MSP: methylation-specific PCR
