# Supplementary figures and images for: Aberrant expression of GSTM5 in lung adenocarcinoma is associated with DNA hypermethylation and poor prognosis
Source: BMC Cancer. 2022 Jun 21;22:685. doi: 10.1186/s12885-022-09711-0 (PMC9214983; doi:10.1186/s12885-022-09711-0)

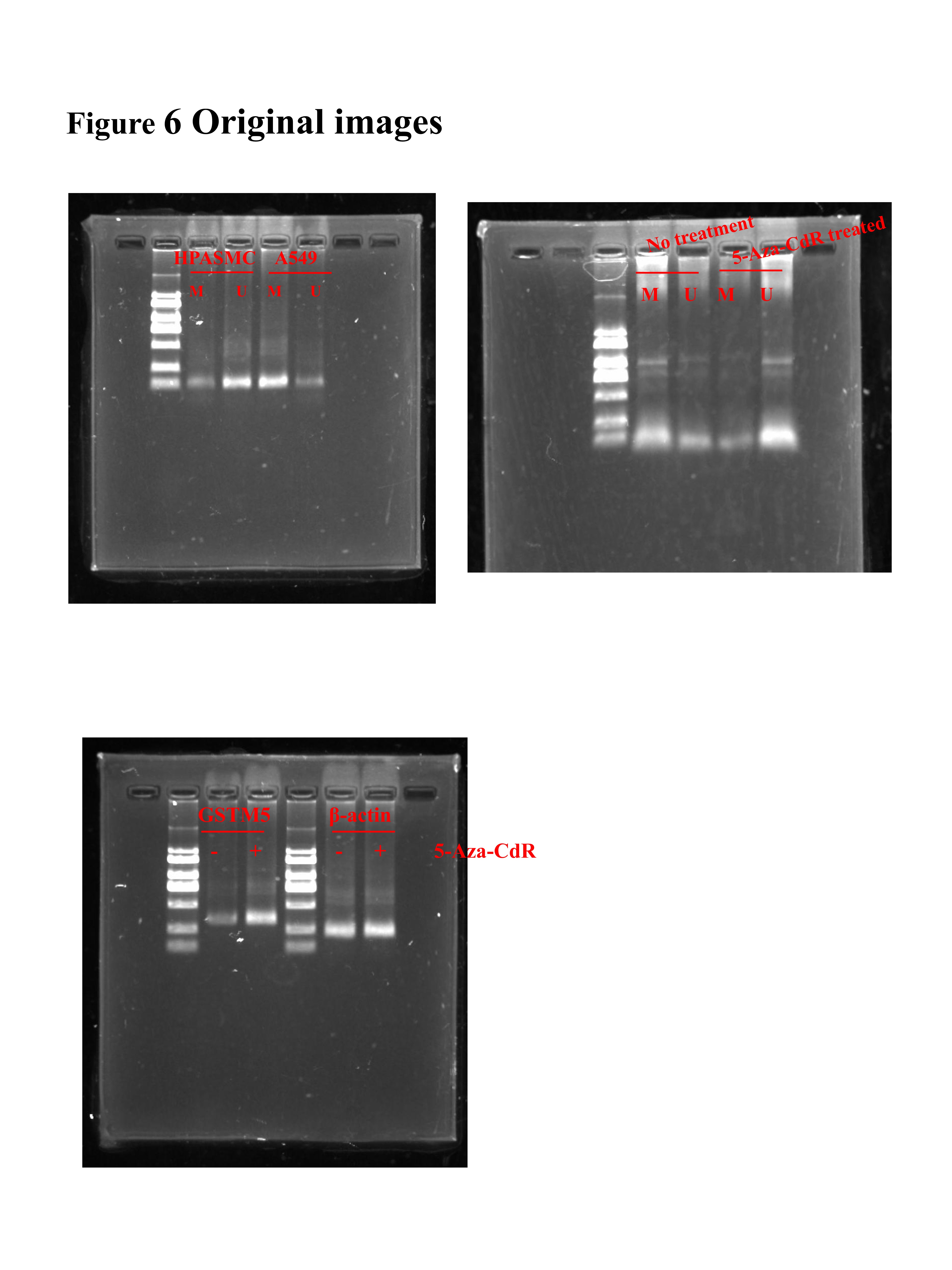

Supplement: Supplementary file 4 — Additional file 4. [file 12885_2022_9711_MOESM4_ESM.jpg]
